# Adrenocortical carcinoma: selective internal radiation therapy and liver metastases

**DOI:** 10.1530/EO-23-0041

**Published:** 2024-07-08

**Authors:** Leo Baxendale-Smith, Karim El-Shakankery, James Gordon-Smith, Lucy Wall

**Affiliations:** 1 Department of Medical Oncology, Edinburgh Cancer Centre, Western General Hospital, Crewe Road, South, Edinburgh, United Kingdom; 2 Department of Interventional Radiology, Royal Infirmary of Edinburgh, Edinburgh, United Kingdom

**Keywords:** adrenal, adrenocortical carcinoma, liver metastasis, selective internal radiation therapy, SIRT

## Abstract

Selective internal radiation therapy (SIRT) is a novel intervention for both primary and metastatic malignant liver lesions. Adrenocortical carcinoma (ACC) is rare with limited treatment options; evidence for SIRT in ACC liver metastases consists of case reports only. Selective internal radiation therapy (SIRT) was employed to treat recurrent liver metastases in a 49-year-old gentleman with ACC, who previously underwent a left-sided hepatectomy. The patient opted for SIRT after reviewing the literature regarding mitotane chemotherapy and its toxicities. Selective internal radiation therapy (SIRT) provided several months of progression-free survival (PFS), with no toxicity and an excellent radiological response. The patient re-presented 12 years after the initial diagnosis with skeletal metastases and sadly died in September 2022. Substantial unmet need exists for effective treatments in ACC, with 75% of patients presenting with incurable disease. Developing widespread disease, SIRT offered 2 years’ PFS in our patient; this was well tolerated with minimal residual liver impairment. Its use in ACC liver-limited disease warrants investigation.

**Significance statement:**

Adrenocortical carcinoma (ACC) is a rare and aggressive tumour with limited treatments. Once metastatic disease develops, existing standard-of-care treatments offer a dismal overall survival, alongside marked toxicities. Selective internal radiation therapy (SIRT) may represent a new intervention in the treatment paradigm for liver-limited, metastatic ACC. Here, we present the case of a patient treated with multiple rounds of SIRT for relapsed, liver-limited ACC, prolonging survival by several years. Recurrent SIRT led to maintained liver function and no toxicities. Little evidence outlines its use in ACC but further study is certainly warranted to ascertain the value of SIRT, considering the limited treatment landscape that currently exists.

## Background

Adrenocortical carcinoma (ACC) is a rare malignancy that typically occurs in the fifth decade of life. Metastatic disease carries a poor prognosis, with overall 5-year survival up to 80% in stage I ACC, but just 13% in stage IV ACC (Ayala-Ramirez *et al.* 2013, Fassnacht *et al.* 2018).

Selective internal radiation therapy (SIRT; often also termed radioembolisation (RE) or trans-arterial radio-embolisation (TARE)) is a form of arterially delivered brachytherapy utilised in the treatment of liver metastases. Importantly, it is associated with limited toxicity in unaffected parts of the liver (Kennedy *et al.* 2015, Boilève *et al.* 2021). There is established evidence regarding the use of β-emitter yttrium-90 (^90^Y) SIRT for liver metastases in neuroendocrine tumours (NETs), with its efficacy and low toxicity profile well discussed (Barbier *et al.* 2016). Literature specific to the use of SIRT for ACC liver metastases is, however, very limited; four case reports (Makary *et al.* 2018, Pigg *et al.* 2020, Lu *et al.* 2021, Lin *et al.* 2022) discuss SIRT in this setting, and one small case series describes increased survival in patients undergoing either SIRT or transcatheter arterial chemoembolization (TACE) (Owen *et al.* 2019).

Despite limited published evidence, due to its low toxicity profile, the 2015 European Neuroendocrine Tumour Society (ENETS) guidelines recommended that SIRT was at least as suitable as TACE for diffuse, liver-limited metastasis from a neuroendocrine primary (Kennedy *et al.* 2015). They also note that, unlike TACE, SIRT is not limited to the number and anatomical location of liver metastases present. That no clear guideline exists for the optimal treatment of liver-limited ACC indicates considerable unmet need in this field.

## Case presentation

A timeline of the case is illustrated in Table 1. In March 2010, a 49-year-old Caucasian male with an unremarkable past medical history consulted his general practitioner with severe hypertension (initially presenting at 260/106 mm Hg). After a 1-month trial of antihypertensives (calcium channel and then angiotensin receptor blockade), without significant clinical benefit, he was referred for a renal ultrasound. A left-sided 7 cm adrenal mass was noted on ultrasound, and the patient was subsequently investigated for an adrenal lesion. Biochemical investigations were most in keeping with a phaeochromocytoma, including borderline-raised urinary normetadrenalines (3.7 μmol/24 h), an unremarkable overnight dexamethasone suppression test, and normal plasma renin and aldosterone levels (all results listed in Table 1). An adrenal-focussed CT was undertaken, confirming the mass was consistent with a phaeochromocytoma.

**Table 1:** Timeline of case report events.

Date	Clinical event
March 2010	49-year-old male presents to the GP with hypertension. Limited response to treatment was reported after an initial 1-month trial of anti-hypertensives (initially 260/106 mm Hg). Secondary hypertension investigations: - USS kidneys – left-sided 7 cm adrenal mass. - 24-hour urine normetadrenaline – 3.7 μmol/24 h, repeat 0.6 μmol/24 h (range: 0.4–3.4). - 24-hour urine metadrenaline – 0.8 μmol/24 h, repeat 0.3 μmol/24 h (range: 0.3–1.7). - Overnight dexamethasone suppression test unremarkable. - CT adrenals – 7.4 × 6.5 × 6.2 cm left adrenal mass, compatible with a phaeochromocytoma. No other abnormality identified. - Nuclear medicine MIBG scan: no evidence of abnormal MIBG uptake, particularly in relation to the abnormal left adrenal gland noted on previous ultrasound/CT. - Plasma renin – within normal range at 3.0 ng/mL/h. - Plasma aldosterone – within normal range at 377 pmol/L; repeat 516 pmol/L.
July 2010	Left adrenalectomy for assumed phaeochromocytoma. Post-operative histology suggestive of an adrenocortical carcinoma: - Weiss criteria – positive features – sample contains less than 25% clear cells; significant nuclear pleomorphism; evidence of capsular invasion; extension into the adjacent peri-adrenal fat; the tumour cells involve vascular sinusoids; single focus highly suspicious for invasion of the tumour cells into a larger blood vessel. - Mitotic rate = 2 mitoses per 50 high power fields (×40). - Immunohistochemistry – tumour cells express melan A and calretinin; no expression of chromogranin or reaction with an antibody cocktail to cytokeratin. Where the percentage of pleomorphic cells is increased, a higher proportion of tumour cells are in active cell cycle (as indicated by the number of cells reacting with the Ki67 antibody). Subsequent radiotherapy to adrenal bed, following positive margins on histology.
January 2014	Liver metastases identified on MRI in segments II, III, IVa, and IVb. Sized 52 mm in segment IVa and 12.5 mm in segment IVb, with the remaining metastases smaller than 10 mm. MRI abdomen, CTCAP, and FDG PET all undertaken pre-resection at the point of relapse. Left hepatectomy was undertaken. Adjuvant mitotane was declined by patient choice, due to toxicity profile.
March 2015	MRI liver, CT liver with contrast, and CT chest undertaken. Arterially enhancing lesions were noted in liver segments V and VII (8 mm and 9 mm in size, respectively). Radiofrequency ablation undertaken.
May 2018	Further liver metastases noted at MDT in segments VI and VII (sized 40 mm; Fig. 1). SIRT discussed with patient and offered due to low toxicity profile.
July and August 2019	FDG PET,CTCAP and MRI abdomen were undertaken, with segment VI and VII lesions re-imaged (42 mm and 70 mm in size, respectively). First round of SIRT was offered and well tolerated. Subsequent round of SIRT offered.
February 2020	Post-SIRT imaging shows a ‘marked response’ to liver lesions, defined by the radiology team as a marked reduction in the size of the segment VII lesion to 34 mm. Although MRI of the liver also notes a slight reduction in the size of segment VI lesion, the very high T2 signal is suggestive of necrosis and response. Liver function unchanged.
November 2020	Disease progression noted near the previously noted segment VI lesion (five subcapsular lesions, including a 40 mm and 26 mm deposit).
January and February 2021	Multiple rounds of SIRT.
April 2021	‘Size reduction response’ in all lesions post SIRT, but residual arterial enhancement was noted in segment VI lesion (30 mm diameter). Mindful of no clear arterial supply to this lesion, transarterial chemoembolisation (TACE) offered for this lesion.
October 2021	TACE undertaken.
February 2022	Disease progression in liver segments VII and VIII (Fig. 2). MRI found disease progression in segment VIII of the liver with a conglomerate of metastases forming a 55 mm mass adjacent to the previously treated lesion, as well as avidly enhancing lesions in segment VII of the liver. Further round of SIRT provided.
May 2022	Ablation of residual disease.
June 2022	Presents with paraplegia following spinal and base of skull metastases.
September 2022	Patient dies aged 61, 12 years after the initial diagnosis and 9 years after the diagnosis of metastatic disease.

In July 2010, laparoscopic left-sided adrenalectomy was undertaken. Post-operative pathology identified an adrenocortical carcinoma, as evidenced by the fulfilment of more than three of the Weiss criteria (Table 1). The patient had a Weiss score of six in total; these positive features included: the sample contains less than 25% clear cells; significant nuclear pleomorphism; evidence of capsular invasion; extension into the adjacent peri-adrenal fat; the tumour cells involve vascular sinusoids; and a single focus highly suspicious for invasion of the tumour cells into a larger blood vessel. Considering the above, the patient had an ESNAT Stage III (T3N0M0) tumour, with the histology commenting on multifocal invasion into the adjacent fatty tissue. Immunohistochemistry was positive for Melan-A and calretinin, and negative for chromogranin and cytokeratin, supporting the diagnosis of an ACC over phaeochromocytoma. Histology of the primary resected lesion did not suggest that a composite tumour was present. As the histology also revealed positive margins, adjuvant radiotherapy was undertaken (45 Gy in 25 fractions) to the left adrenal bed. Radiotherapy was completed in April 2011 and was well tolerated, with no reported toxicity.

Following this, the patient underwent 6-monthly cross-sectional CT imaging for 24 months, followed by an annual MRI from 24 months onwards. Follow-up occurred through the joint neuroendocrine clinic (endocrine and oncology specialties). In January 2014, hepatic metastases were reported in segments II, III, IVa, and IVb, as well as one immediately anterior to the inferior vena cava. Multiple imaging modalities were undertaken, suggesting this was a liver-limited relapse (Table 1). Considering his favourable Eastern Cooperative Oncology Group (ECOG) performance status, he was referred for an extended left hepatectomy, which was performed without complication. Histology of the resected liver metastases reported that the range of histological appearances of the tumours was very similar to those identified in the original resection specimen from 2010, in keeping with metastatic adrenocortical carcinoma. After consideration of adjuvant mitotane, the patient opted against this treatment on the grounds of its toxicity profile, as described in the literature. After hepatectomy, the patient was followed up with a 6-monthly MRI of the liver and CT of the chest, abdomen, and pelvis (CAP) for the 1st year, followed by a 6-monthly CTCAP.

MRI follow-up imaging in March 2015 subsequently identified two arterially enhancing liver lesions in segments V and VIII, for which radiofrequency ablation (RFA) was undertaken in May 2015, with good effect.

**Figure 1:**
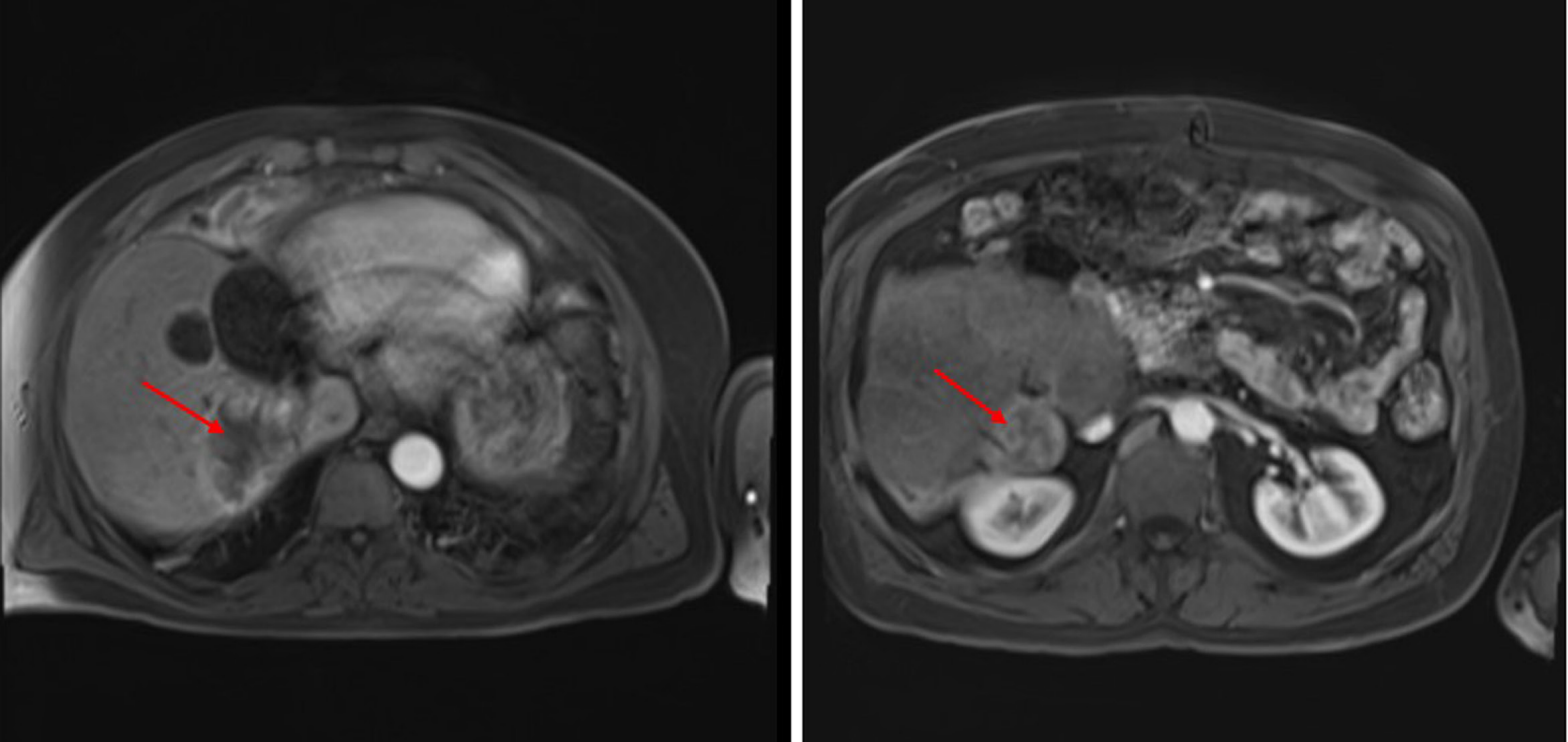
Recurrence in liver segments VI and VII (February 2019).

Three years later, CT follow-up showed two further liver lesions in segments VI and VII. Further imaging, including FDG PET, confirmed the absence of malignancy elsewhere (Table 1). Given the previous extensive liver resection, these were not deemed suitable for further surgical intervention. As he remained well, with a good ECOG performance status, and was asymptomatic of his disease at this point, time was taken to consider the next steps. Fig. 1 shows these liver lesions when re-imaged in February 2019. With the patient having again opted against mitotane, discussion around the management of these latest metastatic lesions considered the use of SIRT and TACE; based on its low toxicity profile, SIRT was opted for to treat his neuroendocrine tumour liver metastases (NETLMs).

^90^Y microsphere (TheraSphere™) SIRT was first undertaken in July 2019 for both lesions. This was well tolerated with no noted toxicities. Post-SIRT MRI was undertaken in December 2019, showing a marked response, with a resultant watch-and-wait policy advised.

Repeat imaging in November 2020 showed recurrent active disease in segment VI. Selective internal radiation therapy (SIRT) (again, ^90^Y microsphere, TheraSpheres™) was again performed in January 2021, mindful of his ongoing liver-limited disease. A multidisciplinary team discussion in April 2021 agreed that SIRT had produced a size reduction response in all lesions; however, as arterial enhancement persisted in the segment VI lesion, TACE was undertaken in October 2021. There was no evidence of a subsequent response to this treatment.

**Figure 2:**
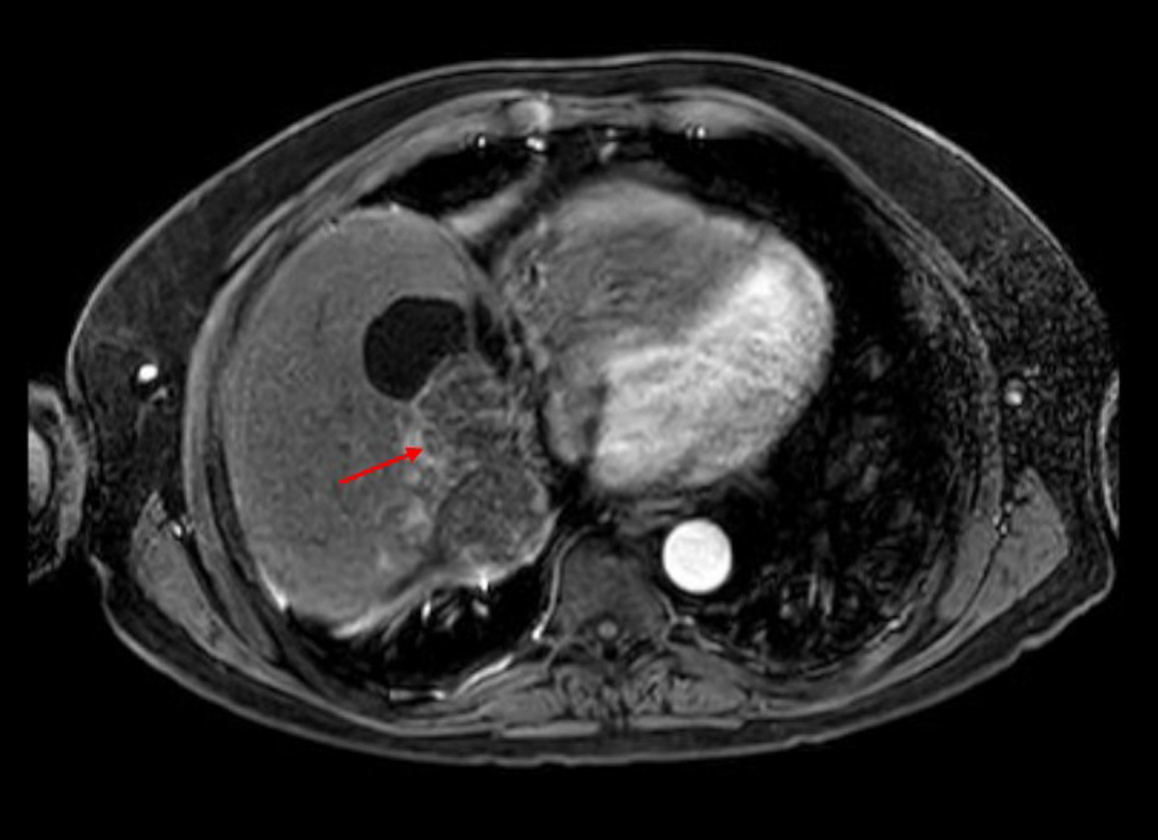
Progression in bland embolisation sites, complete response in SIRT/ablation sites (January 2022).

In February 2022, progression was noted in segments VII and VIII (Fig. 2), for which further SIRT (as before) was undertaken in March 2022, alongside RFA to the same site in May 2022, neither of which produced a notable radiological response.

In June 2022, the patient presented with spinal and base of skull metastatic disease and passed away in September 2022, 12 years after the initial diagnosis and nine years after the diagnosis of metastatic disease.

## Discussion

Adrenocortical carcinoma (ACC) is a rare malignancy with an incidence of one to two cases per million population per year. As such, treatment options are limited, and extensive study proves challenging, with most being retrospective case series. Median overall survival with ACC has been reported as less than 3.2 years, largely attributed to the commonly advanced stage of disease at presentation (Ayala-Ramirez *et al.* 2013). Using the European Network for the Study of Adrenal Tumours (ENSAT) staging system, our patient initially presented with stage II ACC (tumour greater than 5 cm in size with no local invasion or lymph node spread). The median survival for ENSAT stage II ACC has been quoted at 6.1 years (Ayala-Ramirez *et al.* 2013); his treatment (including SIRT) provided him 12.5 years from initial diagnosis, as discussed above.

In all previously reported cases of ^90^Y SIRT use in liver metastases, systemic chemotherapy has also been employed, most commonly in the form of mitotane with (Makary *et al.* 2018, Pigg *et al.* 2020, Lin *et al.* 2022) or without (Lu *et al.* 2021) etoposide, doxorubicin, and cisplatin (EDP). Several of these cases report a complete radiological response to liver SIRT, lasting 24 months post treatment (Makary *et al.* 2018, Pigg *et al.* 2020, Lin *et al.* 2022). In one case, a complete pathological response was also reported, as assessed by a consolidatory partial hepatectomy 7 months post SIRT (Lu *et al.* 2021). Aside from one patient experiencing a post-SIRT adrenal crisis, managed with steroid therapy, treatment toxicity was not reported to be a concern in all other case reports (Lu *et al.* 2021). The most reported standard adverse effects following SIRT are transient transaminase increases, fatigue, nausea, and pain (Kuei *et al.* 2015, Barbier *et al.* 2016, Lu *et al.* 2021).

This case does not represent a cortisol-secreting ACC, as indicated by normal dexamethasone suppression and urinary metanephrines during the initial investigation. The existing literature on SIRT use in ACC is very limited, not only on whether the ACC treated with SIRT was cortisol-secreting (Lin *et al.* 2022), or not (Makary *et al.* 2018), but also on whether cortisol levels were investigated at primary presentation (Pigg *et al.* 2020, Lu *et al.* 2021). This scarcity of evidence further highlights the need for investigation into the use of SIRT in ACC.

The patient discussed in our case opted against mitotane in both the adjuvant and metastatic settings due to its known toxicity profile relative to its benefit (Araújo & Bugalho 2021). At the time of relapsed disease, SIRT appealed to him due to its reported low toxicity and good response in other NETLMs (Kuei *et al.* 2015, Barbier *et al.* 2016). Though recommended by the European Society for Medical Oncology in the metastatic setting, mitotane response rates are low (13–35%), and the data supporting its use is limited. Even with the addition of EDP chemotherapy, employed for more widespread disease, progression-free survival (PFS) gains are modest at best, and no survival advantage has been shown. Considering this, alongside the consistent findings reported elsewhere in support of SIRT, we advocate its use in liver-limited metastatic ACC to produce favourable clinical and radiological effects (Fassnacht *et al.* 2012, Fassnacht *et al.* 2018, Fassnacht *et al.* 2020, Lin *et al.* 2022).

All of the patient’s hepatic metastases, throughout his disease process, demonstrated avid arterial enhancement and washout, making them good candidates for trans-arterial radioembolisation. In this case, SIRT was initially undertaken in July 2019. Its use heralded no toxicity and a 16-month PFS. When further progression was noted, SIRT was carried out again on the grounds of its previous success. This further round of SIRT provided a further 9 months without disease progression. In comparison, PFS in liver-limited ACC has consistently been quoted as less than 9 months when chemotherapy (mitotane +/− further agents) has been utilised (Fassnacht *et al.* 2012). Comparing this to our case suggests that SIRT has a valuable place in the treatment of metastatic liver disease in ACC and warrants further investigation.

Selective internal radiation therapy (SIRT) has been recommended as a treatment modality in both colorectal carcinoma (CRC) and hepatocellular carcinoma (HCC) already. In HCC, SIRT use was shown to be at least equivalent to oral sorafenib with regard to PFS and overall survival but was far better tolerated (Reeves *et al.* 2023). In CRC with liver metastases, its use is typically employed for patients unable to tolerate chemotherapy and those with chemotherapy-resistant disease (Schmoll 2017). Again, in such scenarios, local control did not translate to a PFS benefit, even in those with liver-limited metastatic disease. However, due to the heterogeneity of different solid cancers and the populations they occur in, drawing comparisons in PFS between different cancers should be undertaken with caution.

The use of mitotane in combination with locoregional therapies is discussed in the literature, with European guidelines advocating for its use in ENSAT stage IV low-tumour-burden disease and indolent ACC (Fassnacht *et al.* 2020), due to good reported disease responses (Boilève *et al.* 2021, Roux *et al.* 2022). Despite this background of evidence, the patient discussed in our case report opted to proceed without adjuvant mitotane due to its known toxicity profile (Araújo & Bugalho 2021).

The application of TACE in primary liver tumours and colorectal liver metastatic disease is well discussed in the literature. Though evidence of its use in ACC is comparably lacking and limited to retrospective studies with small sample sizes, TACE has been reported as an effective treatment for ACC-associated liver metastatic disease. Reported cases generally describe its concurrent use with systemic chemotherapies. Evidence suggests its use leads to an acceptable safety profile and decreases in tumour size, alongside improved time-to-progression when used in combination with mitotane (Deng *et al.* 2023) or other agents (Kimpel *et al.* 2024). The use of TACE *or* SIRT in a small series of patients has been reported, and both associated with improved outcomes (Owen *et al.* 2019). Although SIRT and TACE modalities have been directly compared when used in other malignancies (Brown *et al.* 2023, Adriana *et al.* 2023), no literature directly comparing the two in ACC has been published.

Considering the wider literature, no randomised trials or prospective data exist in this setting, further limiting the availability of valuable evidence. The rarity of ACC, let alone liver-limited metastatic ACC, will limit its generation. Our case report is also only the second in the literature to describe SIRT in a patient without metastatic disease at initial presentation. Whilst the patient discussed in our case presented with metastatic disease some 41 months after adrenalectomy and radiotherapy, the time to metastatic presentation after adrenalectomy (stage II ACC) was reported as just 9 months in the only other relevant case (Lin *et al.* 2022). The authors strongly stress the need for more comprehensive literature on relapsed ACC to improve our understanding of this rare condition.

## Conclusion

To our knowledge, this is the 5th reported use of ^90^Y SIRT for liver metastases in ACC. It is, however, the first to have employed no chemotherapy at all. Overall, SIRT provided 21 months’ PFS across two rounds of treatment, both of which were well tolerated. Its application provided excellent clinical and radiological responses without marked toxicity, indicating that its use certainly warrants further study in the liver-limited ACC setting.

## Declaration of interest

The authors declare that there is no conflict of interest that could be perceived as prejudicing the impartiality of the study reported.

## Funding

This work did not receive any specific grant from any funding agency in the public, commercial, or not-for-profit sector.

## Patient consent

Written informed consent for the publication of clinical details was obtained from the patient.
